# Layout Optimization of the Six‐Axis Industrial Robot Based on an Improved Whale Algorithm for Reducing Energy Consumption in Industry 5.0

**DOI:** 10.1002/EXP.20240120

**Published:** 2025-12-22

**Authors:** Kaiyue Cui, Yixiong Feng, Zhaoxi Hong, Zhiwu Li, Fathollahi‐Fard Amirmohammad, Zengwei Ji, Jianrong Tan

**Affiliations:** ^1^ School of Mechanical Engineering Guizhou University Guiyang China; ^2^ State Key Laboratory of Fluid Power and Mechatronic Systems Zhejiang University Hangzhou China; ^3^ Ningbo Innovation Center Zhejiang University Ningbo China; ^4^ Institute of Systems Engineering Macau University of Science and Technology Macau China; ^5^ School of Business Administration Laurentian University Sudbury Ontario Canada

**Keywords:** energy conservation, Industry 5.0, layout optimization, six‐axis industrial robot, whale algorithm

## Abstract

In the advent of Industry 5.0, the harmonious integration of human ingenuity and robotic precision in complex work environments is pivotal for sustainable industrial growth. The six‐axis industrial robot, as an essential part of carrying out cyclic pick‐and‐place tasks in Industry 5.0, usually works in an extremely complex working environment. This intricate working environment makes the six‐axis industrial robot difficult to reach the task points effectively, resulting in a lot of energy consumption. This not only impacts productivity but also leads to excessive energy consumption, which stands at odds with the Industry 5.0 principles of resource conservation. To solve this problem, a novel method to optimize the layout scheme of the six‐axis industrial robot with the goal of minimizing the energy loss is creatively proposed in this paper. First, the reachable workspace and feasible workspace under constraints are mathematically modeled and then obtained. Second, the operability and the minimum singular value are utilized to evaluate the energy loss of the feasible workspace. Third, the whale algorithm is designed and improved to obtain the optimal layout scheme of the six‐axis industrial robot. Finally, a case of the recliner's production line with the six‐axis industrial robot (IRB140; ABB) is provided to validate the effectiveness of the proposed method. The results show that after optimization, the optimal layout scheme has been successfully obtained, and the energy loss has reduced from 0.2917 to 0.2309, a decrease of 20.84%, proving that the proposed method can obtain the optimal layout scheme with lower energy consumption.

## Graphical abstract

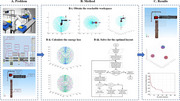

## 1 Introduction

With the development of Industry 5.0, industrial robots have been widely used in the automation line of manufacturing. They play an important role in carrying out cyclic pick‐and‐place tasks in the production line, owing to the advantages of low cost, high safety, and easy supervision [1, 2, 3]. The new report World Robotics 2020 Industrial Robots, which is provided by the International Federation of Robotics, shows a record of 2.7 million industrial robots operating in factories around the world—an increase of 12% compared to 2019. However, it is also exposed in the social perspective that the problem of excessive energy consumption is becoming more and more prominent [4, 5]. Due to the existence of multiple constraints in the working environment [6], industrial robots often have low overall flexibility, which has seriously exacerbated the energy consumption during work. And manufacturers often focus on upgrading the robots themselves, ignoring the impact of the external environment, such as spatial layout. Therefore, it is necessary to take the layout optimization into consideration to reduce the energy consumption of industrial robots during work [2, 7, 8, 9].

The layout optimization of industrial robots is to optimize or adjust the layout of industrial robots according to the actual production. It aims at improving productivity and flexibility, reducing energy consumption, and so on. Pertinently, related research on optimizing the layout concerning for reducing energy consumption has been rapidly gaining extensive attention. Gadaleta et al. [10] established the industrial robot subsystem component to accurately predict the system power and created a cell model in the Dymola environment to optimize the cell layout, thus finding the location of the robot’ base that minimizes the energy consumption. Kouzehgar et al. [11] utilized the four tiling set theorems to define the navigation maps for the robot, and the tiling set theorems are applied to each sub‐area to deliver the best balance between energy and area coverage. Zhang et al. [12] proposed a novel wire‐driven variable‐stiffness joint based on a permanent magnetic mechanism to adjust the joint stiffness through a wide range. This mechanism reduces energy and power to adjust the stiffness, apparently by controlling position and stiffness independently.

Solving the reachable workspace of industrial robots is the primary step in the layout optimization. The reachable workspace is the set of reachable points that the end‐effector of the robot can reach, which is only having connection with the shape and structural parameters of the robot. In order to find the reachable workspace precisely, many scholars have done related research. Rushton et al. [13] proposed a method called the structure atlas to analyze the workspace and identify the reachable kinematic areas of the industrial robots. Gao et al. [14] developed the reachability‐expressive motion planning method to execute the calibration of the robot's reachable space. Ben Lakahal et al. [15] utilized the interval analysis method and interval Taylor series expansion method to obtain the reachable space. What is more, collision detection is introduced to avoid obstacles. The collision‐free reachable workspace is determined by scanning the robot. Terakawa et al. [16] showed the reachable region of slidable‐wheeled omnidirectional mobile robot (SWOM) in omnidirectional movement by discussing the movable range of the sliding joints, and propose a trajectory generation method that enables SWOM to change its movement direction while keeping the reachable region. Zhang et al. [17] adopted the limit boundary searching method and used the surface enveloping theory to obtain the boundary of the single limb space. Su [18] defined all factors that avoid the transitions of the system as unreachable areas in the R‐space, including physical configurations of the system and environmental constraints.

Second, calculating the energy loss is important in the layout optimization. Previous methods for reducing energy consumption focus on trajectory optimization and the energy‐optimal robot selection. And now, a large number of researchers begin to pay attention to the impact of robotic layout. Mohammed et al. [19] calculated the desired forces and torques on the joints and links of the robot to obtain the energy consumption. Paryanto et al. [20] established a dynamic model to analyze the energy consumption. Chen et al. [21] developed a discrete event simulation model to obtain the system energy consumption of the bin‐picking robots and track‐changing robots. Mayor et al. [22] provided an analytical model that predicts the energy consumption, which considers both the tasks and the radio communication. Traditionally, the less flexible the industrial robot is, the more energy consumption the industrial robot has. Meanwhile, some researches reduce energy consumption by decreasing the idle time of industrial robots. In a word, possible strategies to reduce the energy consumption of robotic layout is greatly important for industry.

Obtaining the optimal layout is the last step in the layout optimization. Many studies have investigated intelligent optimization algorithms. For example, Ho et al. [23] proposed an accurate and practical methodology, which is called multi‐objective combinatorial optimization, to optimize the layout of a workstation. Xie et al. [24] verified the effectiveness of the taboo search and non‐dominated sorting genetic algorithm in optimizing the system layout configuration. Kulturel–Konak [25] used the ant colony optimization to determine the best facility layout solution. Aslan et al. [26] made full use of simulated annealing to seek a high‐quality layout scheme with the aim of improving transportation efficiency and reducing congestion risk. Golmohammadi [27] verified the effectiveness of particle swarm optimization, non‐dominated sorting genetic algorithm, and taboo search in solving the layout design of cellular manufacturing systems. Inspired by the unique hunting methods of humpback whales, Australian scholars propose a new meta‐heuristic swarm optimization algorithm, which is called the whale algorithm [28]. The research on utilizing the whale algorithm to solve the optimal layout plan has been paid more and more attention by scholars. Kaveh et al. [29] combined the whale algorithm and colliding bodies optimization to solve the construction site layout planning problem. Zavari et al. [30] utilized the guided population archive whale optimization to optimize layout objectives and consider constraints. Compared with conventional methods, the whale algorithm is an efficient algorithm to solve layout optimization problems, which performs better in convergence speed and balancing exploration and exploitation [31]. Therefore, we attempt to design and improve the whale algorithm for better performance.

According to the literature review, we can know that the reachable workspace of the six‐axis industrial robot can be effectively obtained, and the layout optimization based on the whale algorithm is an acceptable and practical approach. However, the reachable workspace does not comprehensively consider the impact of interference constraints and time constraints, ignoring the impact from the external environment. And the energy consumption of carrying out cyclic pick‐and‐place tasks has not been quantified and visible either. To handle these problems, we discuss the layout design of the six‐axis industrial robot from the perspective of constraint relationships. Based on the constraint relationships, we obtain the reachable workspace and the feasible workspace of the six‐axis industrial robot. To weigh the influence of layout on energy, we utilize the operability and the minimum singular value as evaluation indices to quantify the energy loss, and divide the reachable workspace according to the energy loss. Finally, the optimal layout scheme for the six‐axis industrial robot with the goal of minimizing the energy loss is obtained based on the improved whale algorithm. The contributions of our work are the following:

1. A novel method for layout optimization of the six‐axis industrial robot with the goal of minimizing the energy consumption is proposed. Through this method, the energy consumption during the operation of six‐axis industrial robots can be effectively reduced, which has important practical value and environmental significance.
2. The interference constraints and time constraints of the six‐axis industrial robot are analyzed, and the reachable and feasible workspaces of the six‐axis industrial robot under constraints are creatively obtained. This not only provides a theoretical basis for optimizing the workspace of six‐axis industrial robots, but also provides scientific guidance for practical operations.
3. A calculation method for energy loss during the operation of a six‐axis industrial robot is introduced, which can successfully obtain the energy loss in the task area and divide and visualize the energy in the workspace of the six‐axis industrial robot. This not only makes the evaluation of energy efficiency more intuitive but also provides a new analytical tool for optimizing the energy efficiency of industrial robots.
4. The whale algorithm is designed and improved by introducing the nonlinear convergence factor to obtain the optimal layout of the six‐axis industrial robot, minimizing its energy loss during operation. The improved whale algorithm enhances search efficiency and convergence performance, providing a more effective algorithm tool for solving complex optimization problems.

The rest of this paper is organized as follows. Section 2 establishes the mathematical models of the reachable workspace and the feasible workspace under constraints of the six‐axis industrial robot. Section 3 analyzes the method to calculate the energy loss and divides the reachable workspace based on the energy loss. Section 4 introduces the principles of the improved whale algorithm and layout optimization. Section 5 takes the six‐axis industrial robot (IRB140; ABB) as an example to verify the effectiveness of the proposed method. Section 6 concludes this work and describes some development prospects.

## 2 Mathematical Model

The reachable workspace and the feasible workspace of the six‐axis industrial robot are extremely necessary to obtain, which provide the basis for the subsequent layout optimization. The reachable workspace can be obtained first, and then the feasible layout workspace can be obtained after considering the interference constraints and other scenarios. Industrial robots usually do not have to strictly follow a continuous trajectory to perform cyclic pick‐and‐place tasks. In addition to the pick‐up point and the placement point, it is generally necessary to specify several interpolation points that the robot must pass according to the actual production situation.

**FIGURE 1:**
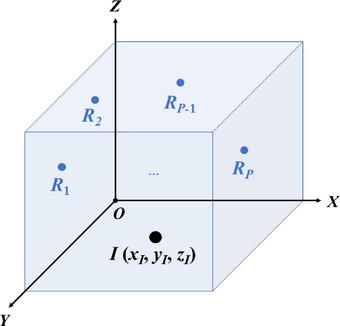
The Cartesian workspace of the six‐axis industrial robot to complete the cycle handling task.

We only consider the situation where the layout of the six‐axis industrial robot is parallel to the floor of the workshop. We assume that the pick‐up point (i.e., start point) and the placement point (i.e., terminal point) of the six‐axis industrial robot are fixed. In addition to these two points, several predefined Cartesian workspace points are still required. The Cartesian coordinate system {*O*} is set up, whose *XOY* plane is parallel to the factory's ground, and the positive direction of the *Z* axis is vertical upward. The Cartesian workspace points that must pass are denoted as *R_p_*, *p*∈[1, *P*], and the center of the base of the industrial robot can be set as *I* (*x_I_*, *y_I_*, *z_I_*), as shown in Figure 1.

### 2.1 The Mathematical Model of the Reachable Workspace

The reachable workspace of the six‐axis industrial robot is a collection of Cartesian workspace points that the robot's end‐effector can reach. This set is only related to the structural parameters, kinematics constraints, the geometric structure size of the robot, and the limit angle that each joint can rotate. In general, the reachable workspace of the six‐ axis industrial robot only considers the position that the robot's end‐effector can reach without considering the posture to reach this point.

Owing to the difference in the rotational range of each joint and the requirement for the forward kinematics derivation, the reachable workspace is the numerical workspace mapped from a multi‐dimensional feasible workspace composed of the angles of each joint. The mathematical model is shown in Equations (1)–(4):

(1) $$\Omega =\underset{\forall q_{a}\in Q}{⋃}\left\{E_{a}\right\}$$

(2)

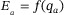

(3) $$Q=\left\{\theta |\forall i\in \left[1,n\right],\theta_{i}\in \left[\theta_{\min,i},\theta_{\max,i}\right]\right\}$$

(4) $$\theta ={\left[\begin{array}{ccc} \theta_{1} & \;\text{…} & \;\theta_{n} \end{array}\right]}^{T}$$

where *n* represents the number of joint variables, *Q* represents the set of joint variables, and *f*(*q_a_*) represents the Equation for calculating the terminal point of the industrial robot in the Cartesian workspace when the joint variable is *q_a_*. *q_a_* is the joint variable of the six‐axis industrial robot. *E_a_* represents the Cartesian workspace point corresponding to its end actuator.

In this paper, the Monte Carlo method is used to solve the reachable workspace of the six‐axis industrial robot under unconstrained conditions. The Monte Carlo method is a numerical simulation method based on probability statistics, which is also called a statistical simulation method [32]. It deals with or fits complex mathematical problems that are difficult to solve by constructing regular random variables. The random sampling points produced by the Monte Carlo method can simulate the corresponding rotation angle of each joint of the six‐axis industrial robot. When the number of sampling points is large enough, the set of points can approximately represent the reachable workspace of the robot. Based on Zhao et al. [15], considering the insufficient uniformity of pseudo‐random numbers used by classical Monte Carlo, we use the *Sobol* sequence of quasi‐random numbers with a better uniformity as the random number generator and the *n*‐dimensional quasi‐random number *Sobol* sequence generated by MATLAB can be written as *S*_(_ *_n_*_)_, as shown in Equation (5), in which each element is randomly selected between [0,1]. The rotation angle of the *i*th joint of the robot can be expressed as Equation (6).

(5) $$S_{\left(n\right)}=\left[\begin{array}{ccc} s_{1} & \text{…} & s_{n} \end{array}\right]$$

(6) $$\theta_{i}=\theta_{\min,i}+\left(\theta_{\max,i}-\theta_{\min,i}\right)\cdot S_{\left(n\right)}\left(i\right)$$

The detailed process of solving the reachable workspace of the six‐axis industrial robot under unconstrained conditions based on the Monte Carlo method in MATLAB is as follows:

1. The Denavit–Hartenberg (DH) parameters of the six‐axis industrial robot are initialized;
2. A set of random numbers is generated based on the quasi‐random numbers, Sobol sequences, and the rotation angle of each joint is obtained;
3. Based on the forward kinematics mentioned above, the position of the end‐effector of the six‐axis industrial robot is obtained. The values and calculation times are recorded. If the number of calculations does not reach the preset value, repeat the above process; otherwise, the recorded value is output as a set. The set output is the approximate reachable workspace of the six‐axis industrial robot in the Cartesian coordinate system without constraints.

**FIGURE 2:**
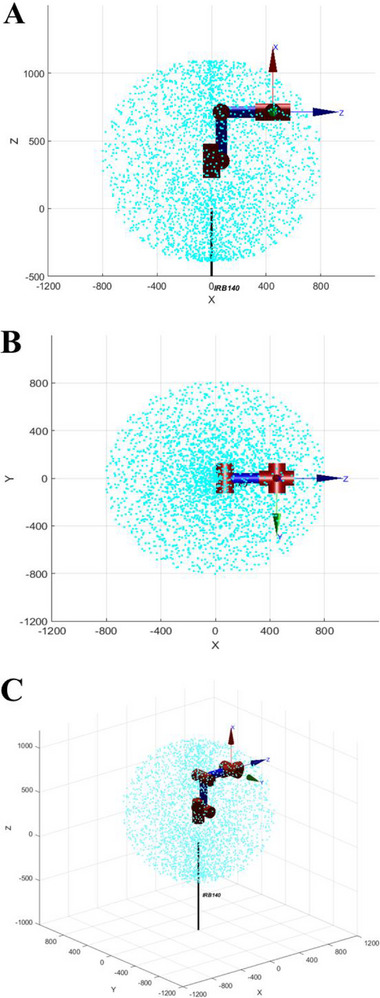
The simulation of the reachable workspace of the six‐axis industrial robot (IRB140; ABB). (A) XOZ plane. (B) XOY plane. (C) Axonometric drawing.

According to the above method, 3000 groups of random number Sobol sequences are taken to approximate the reachable workspace. The reachable workspace obtained by simulation in MATLAB is shown in Figure 2. The limit values of joints’ rotation angles of the six‐axis industrial robot (IRB140; ABB) are shown in Table 1.

**TABLE 1:** Limit values of joints’ rotation angles of the six‐axis industrial robot (IRB140; ABB).

Jointno.	1	2	3	4	5	6
Maximum angle ${(}^{\circ })$	180	110	50	200	120	400
Minimum angle ${(}^{\circ })$	−180	−90	−230	−200	−120	−400

### 2.2 The Mathematical Model of the Feasible Workspace Under Interference Constraints

The feasible workspace is the area where the end‐effector can perform tasks under constraints. The feasible layout of the six‐axis industrial robot generally needs to consider the following interference constraints when carrying out cyclic pick‐and‐place tasks.

1. The base of the robot must be placed above the horizontal plane, and the height of the base is lower than the lowest horizontal height of the necessary interpolation points;
2. The physical interference is caused by the location and size of peripheral devices;
3. Cartesian workspace points in the moving area must be within the actual reachable workspace.

For the physical constraint (1), it can be expressed as Equations (7) and (8):

(7) $$I\left(x_{I},y_{I},z_{I}\right)|0<z_{I}<h_{\min}$$

(8) $${\Omega_{I}}^{\prime }|z>0$$

where *h*_min_ represents the lowest horizontal height of the interpolation points when the center of the base is *I*.

For the physical constraint (2), because the location of peripheral devices is generally determined before the layout of the six‐axis industrial robot, the physical model of each peripheral device can be represented by the method of constructive solid geometry, and then expressed by the data structure of an octree and converted to the cloud set corresponding to Cartesian workspace points. Considering that the robot needs to take up quite a bit of workspace, the robot should have a certain safety distance from the cloud sets to prevent the physical interference. For this requirement, *D_j_* is used to represent the point cloud sets of peripheral devices in the Cartesian system {*O*}. The two‐dimensional coordinates of the center *I* of the robot's base are set to (*x_I_*, *y_I_*). The shortest distance between *I* and *D_j_* on the *XOY* plane is set to *s_j_* and the safe distance is set to *ϛ*. Therefore, the constraint corresponding to the physical constraint (2) can be expressed as Equation (9):

(9) $$s_{j}\geq \varsigma$$

The mathematical model of the reachable workspace of the six‐axis industrial robot under this layout can be expressed as Equation (10):

(10) $${\Omega_{I}}^{\prime }=\Omega -\left(D_{1}\cup D_{2}\text{…}\cup D_{m}\right)$$

where *m* represents the total number of peripheral devices other than the pick‐up and placement equipment.

For the physical constraint (3), it can be expressed as Equation (11):

(11) $$\left(R_{1}\cup R_{2}\text{…}R_{P}\right)\subset {\Omega_{I}}^{\prime }$$

where *R_n_* represents a number of Cartesian interpolation points that the robot must pass through when carrying out pick‐and‐place tasks.

Hence, the center *I* (*x_I_*, *y_I_*, *z_I_*) of the six‐axis industrial robot and the reachable workspace *Ω_I_* should satisfy Equations (7)–(11), then the set of the *I* (*x_I_*, *y_I_*, *z_I_*) is the feasible layout of the six‐axis industrial robot under the physical interference constraints.

### 2.3 The Mathematical Model of the Feasible Workspace Under Time Constraints

The feasible workspace of the six‐axis industrial robot under time constraints is actually to find out all the places where the six‐axis industrial robot can be placed so that it can complete the tasks in the specified time. A complete pick‐and‐place task consists of two operations, namely the handling operation and the homing operation. The handling operation represents the process that starts from the pick‐up point, goes through the interpolation points, and finally reaches the placement point. The homing operation represents the process that starts from the placement point, goes through interpolation points, and finally reaches the pick‐up point. Generally, the industrial robot does not have to pass through the interpolation points when it is in the process of a homing operation. The times of the handling and homing operations are fixed values determined by the production beat, which can be set to *t*_O_ and *t*_H_.

Since there is often more than one inverse solution of an industrial robot at a specified Cartesian point, any interpolation point has multiple sets of feasible joint configurations. To perform the desired operation completely within a specified time, the industrial robot needs to find at least one set of joint configurations under the conditions of meeting kinematic and dynamic constraints, so that it can pass through each interpolation point in Cartesian space in turn within a specified time. In addition, except for the pick‐up point and placement point, other interpolation points can be reached at any time, so curve interpolation fitting cannot be used.

Based on the above analysis, it is only necessary to subtract the adjacent angles corresponding to each joint configuration in chronological order, and accumulate the absolute values after subtraction to obtain the total angle of each joint after rotation. Suppose the industrial robot rotates at a uniform speed. If the ratio of the total rotation angle of each joint to its average angular velocity is less than the predetermined time, it means that the desired cyclic pick‐and‐place task can be completed under time constraints [33].

For the handling operation, the industrial robot needs to start from the pick‐up point, go through the interpolation points, and finally reach the placement point. Therefore, the time constraint can be expressed as Equation (12):

(12)

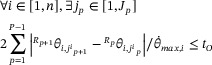

where 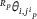 represents the corresponding angle value of the *i*th joint under the *j_p_* th joint configuration at the *p*th interpolation point. ${\overset{\cdot }{\theta }}_{\max,i}$ represents the maximum angular velocity allowed at the *i*th joint. *P* represents the total number of Cartesian workspace interpolation points the six‐axis industrial robot needs to pass through.

For the homing operation, in general, the six‐axis industrial robot does not have to pass through the interpolation points when returning from the placement point to the pick‐up point. Therefore, the homing operation means that the industrial robot needs to start from the placement point and finally reach the placement point. The time constraint can be expressed as Equation (13):

(13)

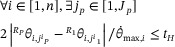




As a result, all the sets of *I* (*x_I_*, *y_I_*, *z_I_*) satisfying Equations (12) and (13) are the feasible workspaces of the six‐axis industrial robot under the time constraints.

When determining the feasible workspace of the six‐axis industrial robot, the reachable workspace under the interference constraints can be obtained first, and then the time constraints can be considered to reduce the amount of calculation.

## 3 Analysis of the Energy Loss

The energy loss is used to describe the energy consumption per unit time during work, which is related to many factors, including flexibility, obstacles, layout scheme, and so on. In general, the stronger overall mobility the robot has, the more flexible the robot is, and the smaller the energy loss will be. At present, the main indicators used to measure the mobility of the six‐axis industrial robot are operability, minimum singular value, and condition number. In this paper, the operability and the minimum singular value are selected to measure the energy loss of the six‐axis industrial robot, which can evaluate the flexibility and energy consumption of each direction in the process of cyclic handling tasks.

### 3.1 Definition and Calculation of the Energy Loss

1. Operability

The operability can measure the mobility of the six‐axis industrial robot in all directions and evaluate the overall energy consumption of the robot. The definition of operability is shown in Equation (14) [34]:

(14) $$\omega =\sqrt{\det\left(J_{F}\left(\theta \right){J_{F}}^{T}\left(\theta \right)\right)}$$

where *J*_F_(*θ*) represents the Jacobian matrix of the six‐axis industrial robot, and det(*J*_F_(*θ*)*J*_F_ *^T^* (*θ*)) represents the determinant of *J*_F_(*θ*)*J*_F_ *^T^* (*θ*).

The singular values of the Jacobian matrix are decomposed by using Equations (15) and (16):

(15) $$J_{F}\left(\theta \right)=U\cdot \Sigma \cdot V^{T}$$

(16) $$\Sigma =\left[\begin{array}{ccccc} \sigma_{1} & 0 & 0 & 0 & \begin{array}{cc} \text{…} & 0 \end{array}\\ 0 & \sigma_{2} & 0 & 0 & \begin{array}{cc} \text{…} & 0 \end{array}\\ 0 & 0 & \ddots & 0 & \begin{array}{cc} \text{…} & 0 \end{array}\\ 0 & 0 & 0 & \sigma_{r} & \begin{array}{cc} \text{…} & 0 \end{array} \end{array}\right]$$

where *U* and *V* are respectively *r *× *r* and *n *× *n* orthogonal matrices. *σ*_1_, *σ*_2_, ⋅⋅⋅, *σ_r_* are the singular values of *J*_F_(*θ*) and *σ*_1_ ≥ *σ*_2_ ≥ ⋅⋅⋅ ≥ *σ_r_* ≥ 0.

The *ω* can be simplified as Equation (17):

(17) $$\omega =\sigma_{1}\sigma_{2}\text{…}\sigma_{r}$$

2. Minimum singular value

The minimum singular value *σ_r_* can measure the mobility of the six‐axis industrial robot in the direction with the worst performance. The smaller the *σ_r_* is, the less flexible the industrial robot is. In general, the minimum singular value at any Cartesian point is between 0 and 1.

Therefore, the flexibility of the six‐axis industrial robot can be expressed as Equation (18):

(18) $$f_{R}=\left(\eta_{1}\cdot \rho \cdot \omega +\eta_{2}\cdot \sigma_{r}\right)$$

where *ρ* is the scaling factor that makes the larger value of *ω* be in the interval [0, 1]. *η*_1_ represents the weight of the operability. *η*_2_ represents the weight of the minimum singular value, and *η*_1_ + *η*_2_ = 1. The weights can be adjusted according to the numerical size and distribution.

Thus, the energy loss can be further expressed as Equation (19):

(19) $$\varpi_{R}=1-f_{R}$$

### 3.2 Division of the Reachable Workspace Based on the Energy Loss

The reachable workspace of the six‐axis industrial robot can be divided according to the energy loss under a certain attitude. The lower the energy loss of the region is, the lower the overall energy consumption will be. The specific steps dividing the reachable workspace are as follows:

1. Preset the parameters required for the calculation, including the posture of the end‐effector and the number of energy loss points;
2. Use the limit angle values of each joint as the upper and lower bounds to produce the *Sobol* random sequences corresponding to the number of preset energy points, and further obtain the corresponding number of joint variables;
3. The Cartesian workspace points of the robot's end‐effector corresponding to different joint variables are obtained from the DH parameters and forward kinematics, and the reachable workspace under unconstrained condition is obtained;
4. For any Cartesian workspace point *R* of the robot's end‐effector, solve the corresponding inverse solution under the *G*_pick_ attitude by the inverse kinematics. If there is no inverse solution, it is considered that *R* is hard to reach under the *G*_pick_ attitude, and then delete the point and continue to calculate the next point. If there are one or more joint configurations, the corresponding Jacobian matrix is calculated with the smallest sum of the absolute values of each joint's rotation angles from the initial motion pose;
5. Calculate the corresponding energy loss of the point based on the Jacobian matrix $\varpi_{R}$;
6. Judge the size of $\varpi_{R}$ and compare it with the preset energy loss threshold *ξ*. If $\varpi_{R}$ is larger than *ξ*, the Cartesian point *G*_pick_ is regarded as the actual inoperable point and return to step (2). Otherwise, the point and its energy loss are stored in the set of energy loss points, and turn to step (7);
7. Determine whether the number of points in the energy loss points reaches the preset number at this time. If not, return to step (2), or end.

**FIGURE 3:**
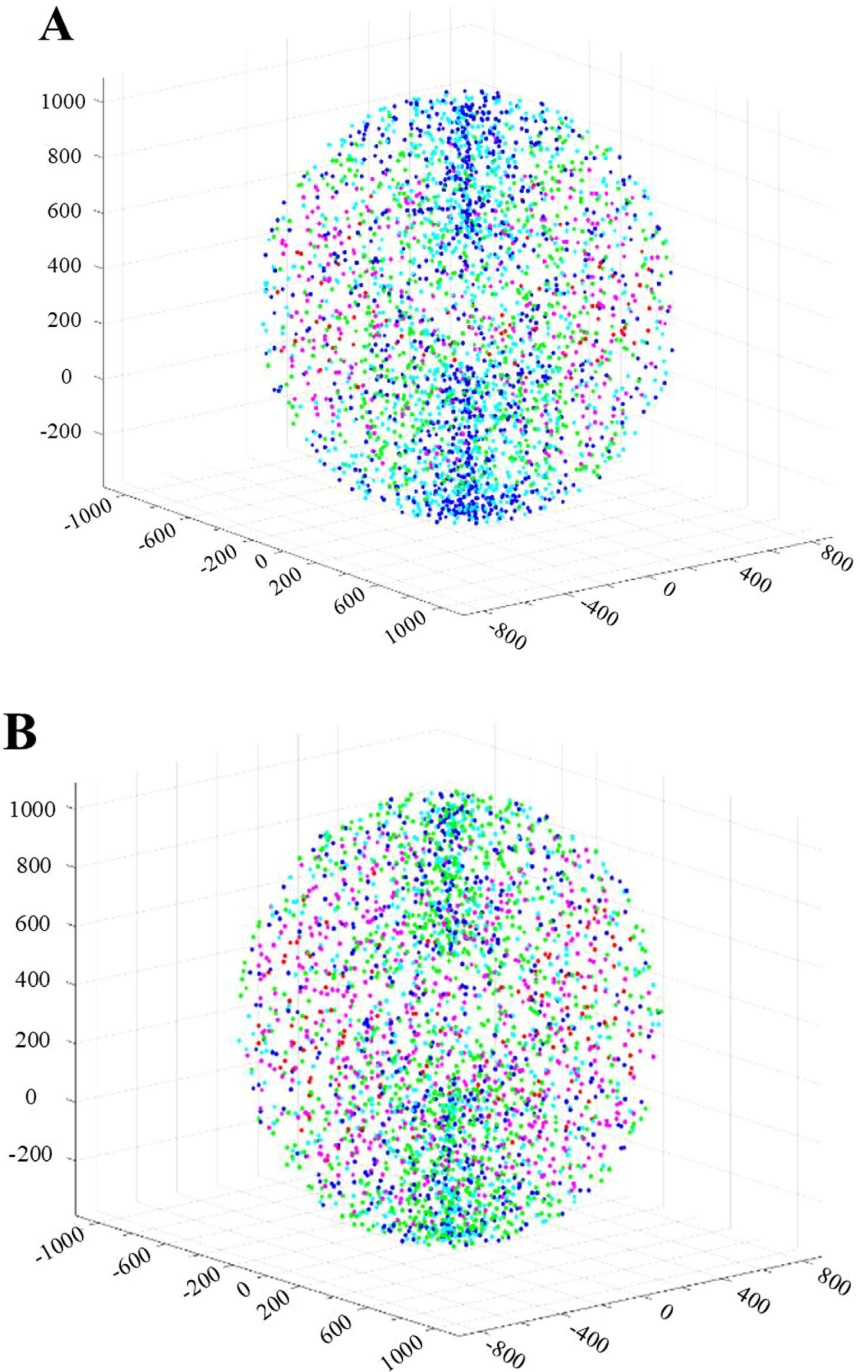
Simulation results of dividing the reachable workspace according to energy loss. (A) The weight of operability is 0.8. (B) The weight of operability is 0.2.

Take the six‐axis industrial robot (IRB140; ABB) as an example. The scaling factor *ρ* is set to 1e‐8. The *Sobol* sequence number is 3000. Figure 3A,B shows the energy loss of each point in the reachable workspace with weights of operability being 0.8 and 0.2, respectively. The visual energy loss threshold is set to 0.02. The energy loss of each point less than 0.2 is marked as red, 0.2–0.4 is pink, 0.4–0.6 is light green, 0.6–0.8 is light blue, and not less than 0.8 is dark blue. If the average energy loss of each point in the reachable workspace is less than 0.4, it is considered that it can cause less energy consumption.

### 3.3 Energy Loss of the Cyclic Handling Task

The area of carrying out the handling task is mainly concentrated in the region composed of each Cartesian workspace interpolation point and the nearby reachable points. Therefore, the area of carrying out the handling task can be regarded as a series of spherical spaces with the interpolation points as the spherical center. The average value of energy loss of these spherical spaces can be approximately represented as the energy loss of the handling task area. Besides, the number of joint configurations at each interpolation point under different layouts also affects the overall energy loss. The more joint configurations the Cartesian point has, the easier it is to be reached by the robot's end‐effector. The more possible the end‐effector is to reach each point in the peripheral space at different times, the higher quality the handling task has.

To this end, an evaluation coefficient is set to evaluate the impact of the number of joint configurations under different layouts on the energy loss. It can be expressed as Equation (20):

(20)

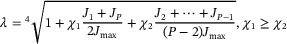

where *J_p_* represents the total number of feasible joint configurations at a specified pose of the *p*th point. *J*_max_ represents the maximum number of feasible joint configurations in the specified attitude of all the accessible points. *x*_1_ and *x*_2_ are respectively the weight coefficients, and *x*_1_ + *x*_2_ = 1. The evaluation coefficient can quantify the influence of the joint configuration of the robot at each interpolation point, especially at the pick‐up point and the placement point. And the coefficient is only slightly greater than 1 and the upper limit is 1.3161 to keep the flexibility within a reasonable range and not be over‐amplified.

The energy loss of each point in the reachable workspace is multiplied by the evaluation coefficient. The expression of the energy loss at any point *R* is shown in Equation (21):

(21)

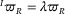




When the center of the six‐axis industrial robot's base is *I* (*x_I_*, *y_I_*, *z_I_*), take the necessary interpolation points *R*_1_, *R*_2_, ⋅⋅⋅, *R_P_* as the centers of the spheres and *δ* as the radius. The spheres are *W*_1_, *W*_2_, ⋅⋅⋅, *W_P_*, and they should satisfy Equation (22):

(22) $$\left(W_{1}\cup W_{2}\cup \text{…}W_{P}\right)\subset \Omega_{I}$$

Take *n_w_* samples of each sphere region. Calculate the energy loss 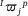 of any sampling point ${r_{j}}^{p}$ in the *p*th sphere region. Therefore, the energy loss of the handling task area can be approximately represented by Equation (23):

(23)

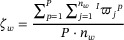




Because $\varpi_{j}\in \left[0,1\right]$ and $\zeta_{w}\in \left[0,1\right]$, the larger *n_w_* is, the closer the energy loss of handling task area is to the actual situation.

## 4 Layout Optimization Based on the Improved Whale Algorithm

### 4.1 The Principle of the Improved Whale Algorithm

Inspired by the unique hunting methods of humpback whales, Australian scholars proposed a new meta‐heuristic swarm optimization algorithm, which is called the whale algorithm [28]. The algorithm simulates the process of a group of humpback whales hunting for prey. The whale algorithm has been widely used in feature selection, image processing, fault diagnosis, and other fields owing to its advantages of fewer adjusting parameters. The optimization process of the whale algorithm can be divided into three stages, which are surrounding the prey, spiral updating, and searching for the prey. The process can be summarized as follows:

1. Consider the group of whales as the primary population generated by random values in the search space;
2. The group of whales move closer to each other according to the individuals in the population that are closer to the prey, and gradually approach the prey in a spiral manner;
3. The group of whales devours the prey by finding clues of the prey. The global optimal solution is obtained when the constraints are satisfied.

In this paper, the whale algorithm is improved by replacing the linear convergence factor with the nonlinear convergence factor, so as to overcome the shortcoming that the original algorithm is easy to fall into a local optimum to some extent. The specific steps of the improved whale algorithm are as follows:

1. Surrounding the prey

At this stage, whales cannot perceive the specific position of the prey. The whale closest to the target prey is taken as the current optimal solution. The other whales in the whale group will update their position according to the current optimal solution, so that the group will gradually move closer to the target prey. The mathematical models of this stage are shown in Equations (24) and (25):

(24) $$D=\left|C\cdot X^{*}\left(t\right)-X\left(t\right)\right|$$

(25) $$X\left(t+1\right)=X^{*}\left(t\right)-A\cdot D$$

where *t* is the current number of iterations. *X* ^*^(*t*) represents the global optimal position of the whale group in the *t*th iteration. *X*(*t*) represents the position of each individual in the whale group in the *t*th iteration. *A* and *C* are the parameters that control the whales to reach the location near the target prey, and their expressions are shown in Equations (26) and (27):

(26) $$A=2a\cdot r_{1}-a$$

(27) $$C=2r_{2}$$

where *r*_1_ and *r*_2_ represent random numbers between [0,1]. *a* is a nonlinear convergence factor that varies with *t*, and it can be expressed as Equation (28):

(28) $$a=2\left[1-\sin\left(\frac{\pi }{2}\cdot \frac{t}{t_{\max\_\mathrm{iter}}}\right)\right]$$

where *t*_max_iter_ represents the maximum number of iterations of the whale group.

2. Spiral updating

At this stage, whales spiral and narrow the encircling circle to hunt. Since the position *X*(*t*) of each individual in the (*t*+1)th generation is between the position *X*(*t*) in the *t*th generation and the global optimal position *X* ^*^(*t*) in the *t*th generation, the mechanism of narrowing encirclement and surrounding the target prey can be simulated by changing the parameter *a*.

The spiral mechanism of whales can be summarized in calculating the distance between each individual and the one which represents the optimal solution at present, and then simulating the real spiral movement of whales to hunt. The mathematical model of this mechanism is expressed as Equations (29) and (30):

(29) $$X\left(t+1\right)=D\cdot e^{bl}\cdot \cos\left(2\pi l\right)+X^{*}\left(t\right)$$

(30) $$D^{\prime }=\left|X^{*}\left(t\right)-X\left(t\right)\right|$$

where *D'* represents the distance between each individual of the whale group and the current optimal solution in the *t*th generation. *b* represents the constant in the spiral movement of the whale group. *l* is the random number between [−1, 1].

In order to realize the parallelism of surrounding the prey and spiral updating, a random number *p* following the distribution of [0, 1] is set. *p* is used to make probabilistic selection of the narrowing encircled mechanism and the spiraling mechanism to update the positions of individuals in the whale group. Assuming that the probability of choosing either mechanism at this stage is equal, the mathematical model is shown in Equation (31):

(31) $$X\left(t+1\right)=\left\{\begin{array}{c} X^{*}\left(t\right)-A\cdot D,p<0.5\\ X\left(t+1\right)=D^{\prime }\cdot e^{bl}\cdot \cos\left(2\pi l\right)+X^{*}\left(t\right),p\geq 0.5 \end{array}\right.$$

3. Searching for the prey

At this stage, the whales will roam randomly throughout the search space in search of the prey. The mathematical model can be expressed as Equations (32) and (33):

(32) $$D_{\mathrm{rand}}=\left|C\cdot X_{\mathrm{rand}}\left(t\right)-X\left(t\right)\right|$$

(33) $$X\left(t+1\right)=X_{\mathrm{rand}}\left(t\right)-A\cdot D_{\mathrm{rand}}$$

where *X*_rand_ represents the position of a random individual in the *t*th generation. When the group uses the random individual's location as a new target, the rest of the whales will move closer to that target.

When the control parameter |*A*| < 1, the search for the local optimal solution will be performed. When |*A*| ≥ 1, the update of group's positions will depend on each other. Therefore, the group can fully search the entire feasible workspace, thereby avoiding the algorithm from falling into the situation of local optimization to a certain extent.

### 4.2 Layout Optimization

In order to minimize the energy loss of the six‐axis industrial robot when carrying out cyclic pick‐and‐place tasks based on the improved whale algorithm, the following settings are made:

1. The variable to be optimized should be the position of the center of the robot's base *I* (*x_I_*, *y_I_*, *z_I_*);
2. The center of the robot's base *I* (*x_I_*, *y_I_*, *z_I_*) and the reachable workspace of the six‐axis industrial robot should satisfy Equations (7)–(13) at the same time;
3. The optimal goal is to minimize the energy loss of the handling task area. It can be shown in Equation (34):
(34) $$\min f=\zeta_{w}.$$

**FIGURE 4:**
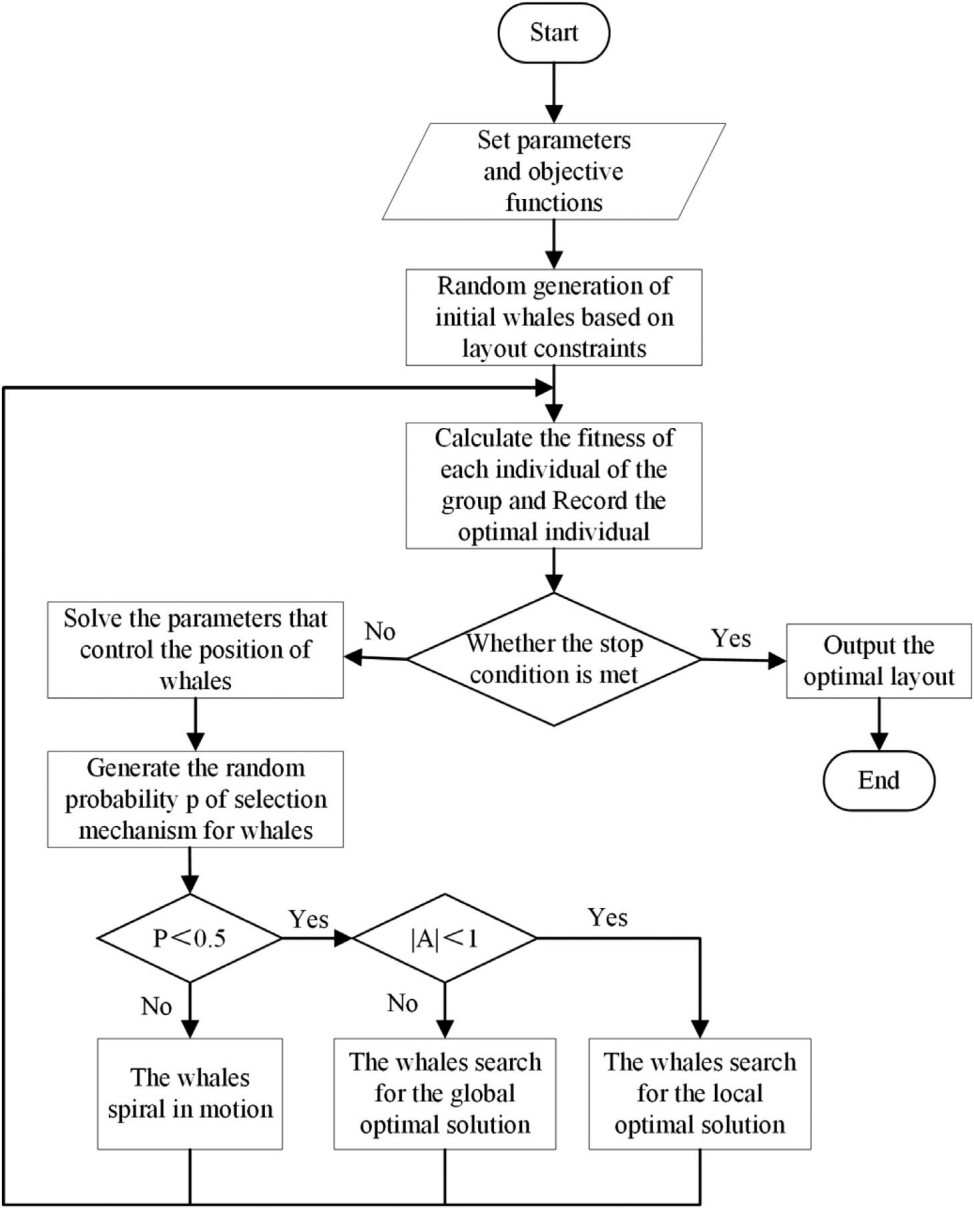
The process of solving the optimal layout based on the improved whale algorithm.

The process of solving the optimal layout of the six‐axis industrial robot is shown in Figure 4. The improved algorithm can ensure reliability while considering the rate of convergence. The layout scheme obtained is usually more environmental than the one based on the unimproved whale algorithm.

## 5 Case Study

To demonstrate the effectiveness of the proposed method, the handling workstation of the six‐axis industrial robot (IRB140; ABB) is applied to design the layout, which is mainly used to produce the recliners of an automobile seat. The production line meets the requirements for the production beat.

**FIGURE 5:**
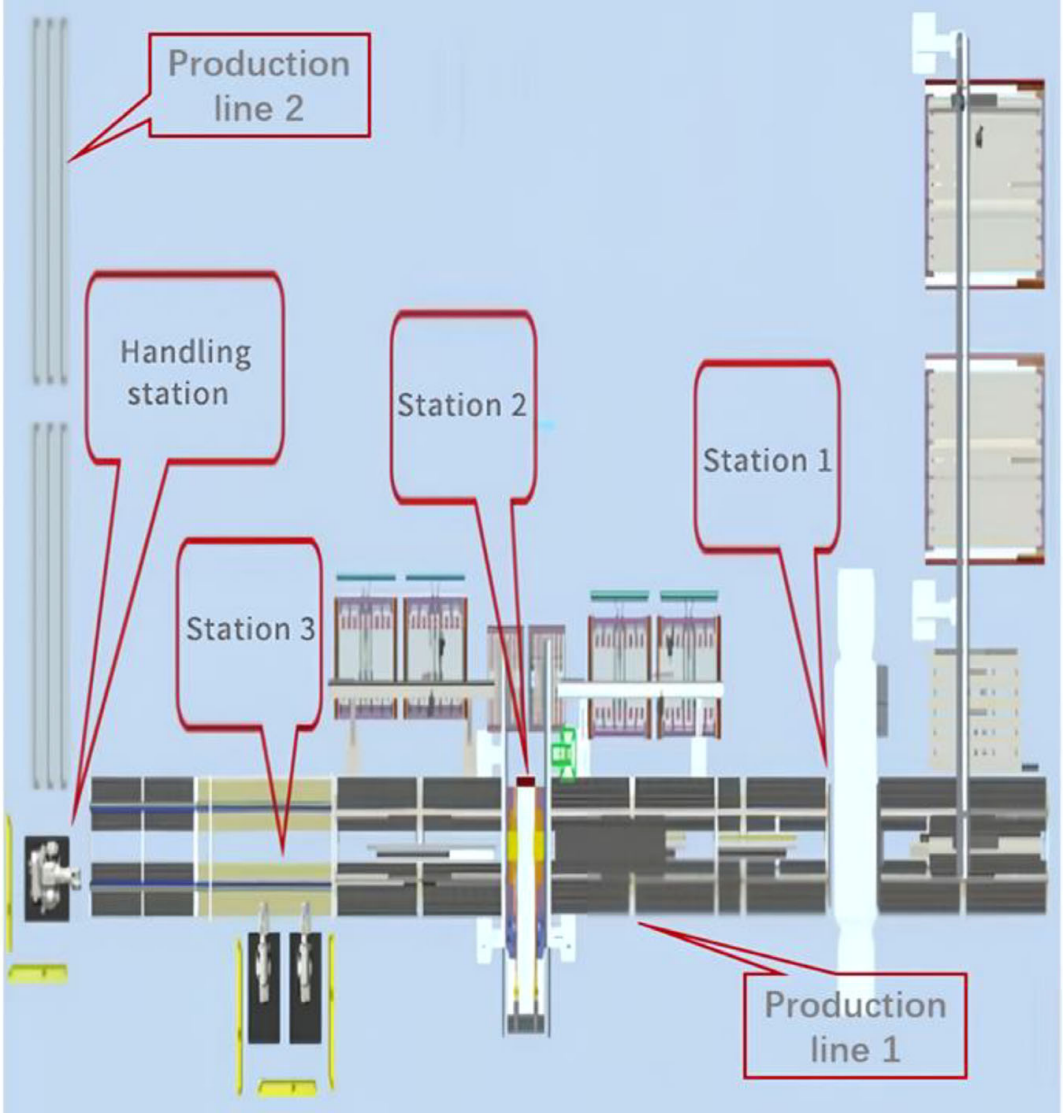
The layout of the recliner's production line.

**FIGURE 6:**
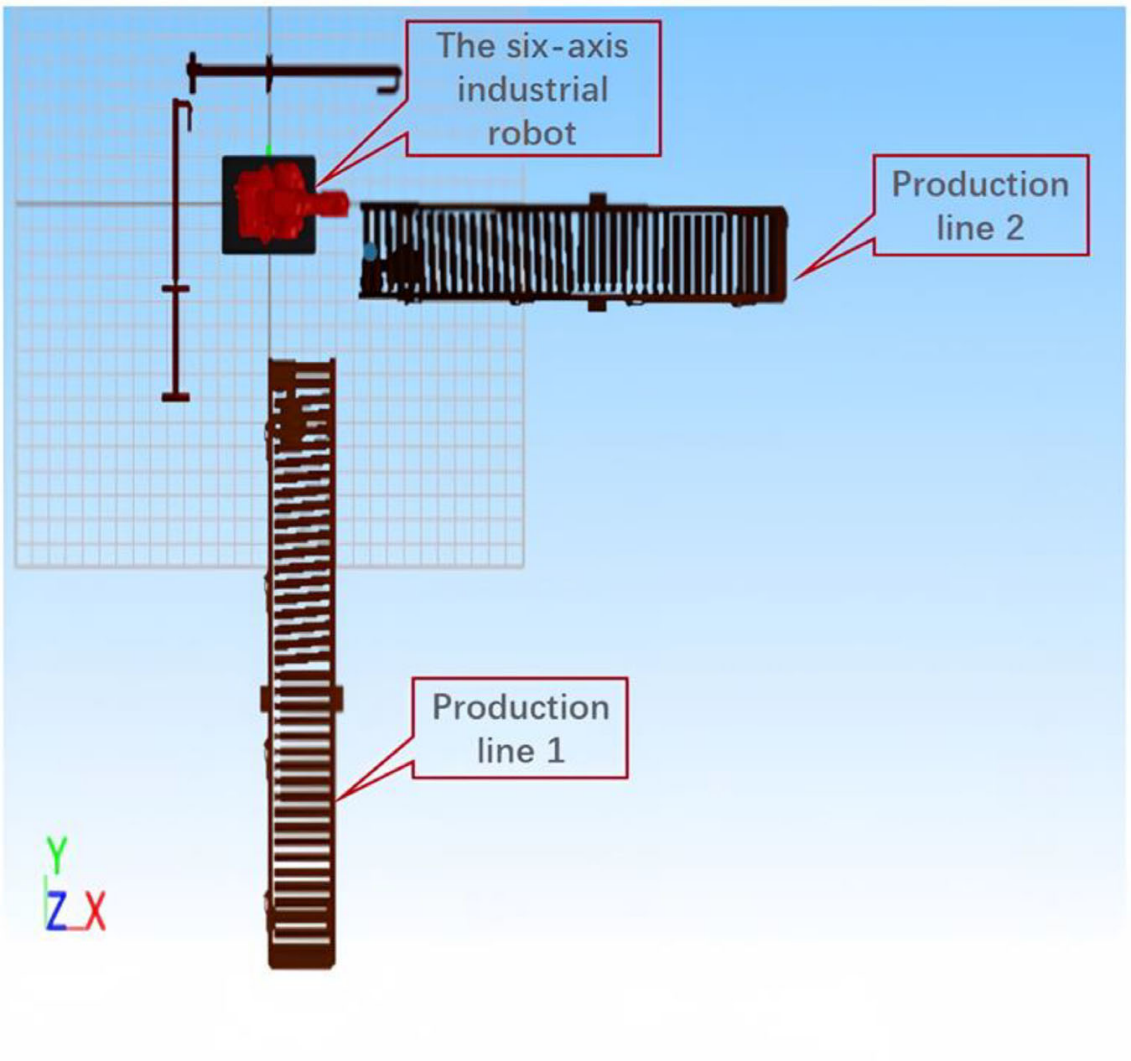
The initial simplified layout of the workstation of the recliner.

The layout of the production line is shown in Figure 5. The recliners of automobile seat and their parts flow from the production line 1 to station 1 for stamping. Then they flow to station 2 for welding, and station 3 for assembly. Finally, this batch of assembled recliners is transferred to production line 2. As the last part of the production line, the assembled recliners need to be moved continuously from production line 1 to production line 2. Import the established 3D model into the RobotApp software. The simplified schematic diagram of the initial layout is shown in Figure 6.

The parameters of the handling workstation are as follows. The geometric center of the robot's base when placed on the platform initially is taken as the origin of the Cartesian coordinate system in millimeters, and the coordinate is (0, 0, 150). The two conveying lines are perpendicular to each other, and are both 4 m in length and 550 mm in width. The highest points of the conveying rollers are respectively 690 and 825 mm from the ground. The horizontal conveying line is 650 mm away from the *Y* axis, and the vertical conveying line is 650 mm away from the *X* axis. The six‐axis industrial robot can be replaced randomly according to the height of the layout, but the area of the platform is a certain value whose length and width are 700 and 500 mm, respectively. The point where the recliner is picked up is in the horizontal conveyor line, and the center coordinate of the bottom is (710, −247, 690). The center coordinate of the bottom where the recliner is placed is (225, 707, 825). The interpolation point that the six‐axis industrial robot must pass to avoid a collision is (313, −49, 1095). The limit bar on the left side of the six‐axis industrial robot is 600 mm away from the *Y* axis, and the limit bar on the upper side is 600 mm away from the *X* axis.

**FIGURE 7:**
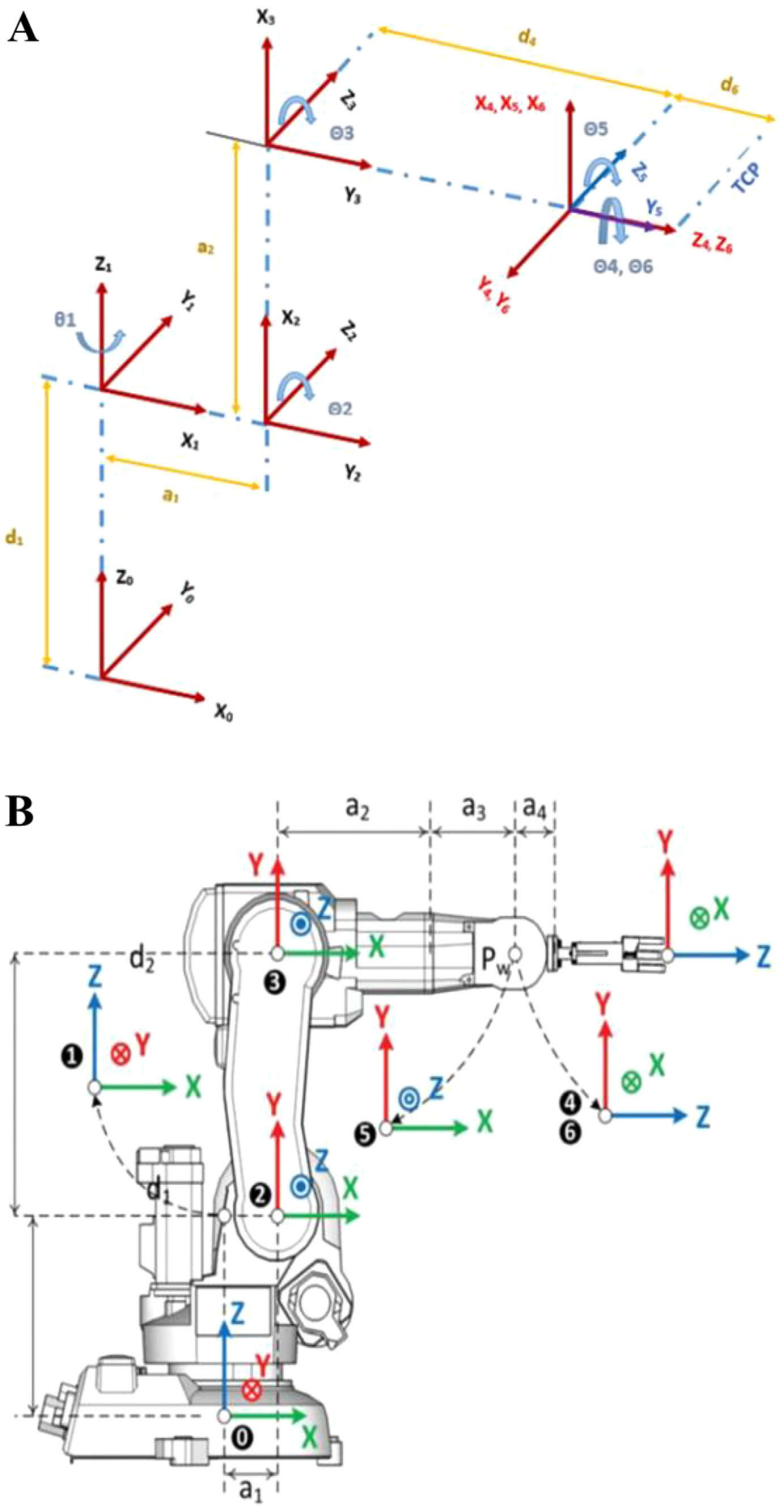
The DH parameter coordinate system of the six‐axis industrial robot (IRB140; ABB). (A) Without the air gripper and (B) with the air gripper.

The DH parameter coordinate system with the air gripper of the six‐axis industrial robot is shown in Figure 7. The air gripper is 150 mm in length. The workpiece to be transported is the recliner of an automobile seat, which is an assembly part with a mass of about 0.5 kg. Its diameter is 100 mm, and the height that can be grasped is 25 mm.

Combined with the dimension parameters of recliners and the air gripper, the coordinates of the start point and terminal point of the end flange's center are respectively (710, −247, 840) and (225, −707, 975). The coordinate at the intermediate interpolation point is (313, 49, 1245).

For the handling workstation of recliners, the variable is the geometric center of the robot's base *I* (*x_I_*, *y_I_*, *z_I_*). The interference constraints are the floor, two conveying lines, and two limit bars. The time constraints are 14 s for handling and 3 s for homing. The three sphere regions *W*_1_, *W*_2_, and *W*_3_ under the *G*_pick_ posture are spheres, whose centers are respectively (710, −247, 840), (225, 707, 975), and (313, −49, 1245) and their radii are all 100 mm. *η*_1_ and *η*_2_ are respectively 0.6 and 0.4. *x*_1_ and *x*_2_ are respectively 0.8 and 0.2. Taking the average of the energy loss of the three sphere regions as the optimization objective, the optimization objective can be expressed as Equation (35),

(35)

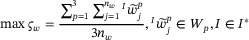

where I^*^ is the feasible location to be optimized.

The safety distance *ζ* is set to 300 mm. The corresponding feasible area I^*^ for the six‐axis industrial robot to perform the cyclic pick‐and‐place task should meet the following Equation (36):

(36) $$\begin{array}{c} \begin{array}{c} I^{*}=\left\{\left(x_{I},y_{I},z_{I}\right)\in D\right\}\\ D=D_{1}\cup D_{2}\cup D_{3}\cup D_{4}\\ D_{1}=\left[-300,350\right]\times \left[-350,300\right]\times \left[0,690\right]\\ D_{2}={\left(x_{I}+70-710\right)}^{2}+{\left(y_{I}+247\right)}^{2}+{\left(z_{I}+350-840\right)}^{2}<700^{2}\\ D_{3}={\left(x_{I}+70-225\right)}^{2}+{\left(y_{I}+707\right)}^{2}+{\left(z_{I}+350-975\right)}^{2}<700^{2}\\ D_{4}={\left(x_{I}+70-313\right)}^{2}+{\left(y_{I}+49\right)}^{2}+{\left(z_{I}+350-1245\right)}^{2}<700^{2} \end{array} \end{array}$$

**FIGURE 8:**
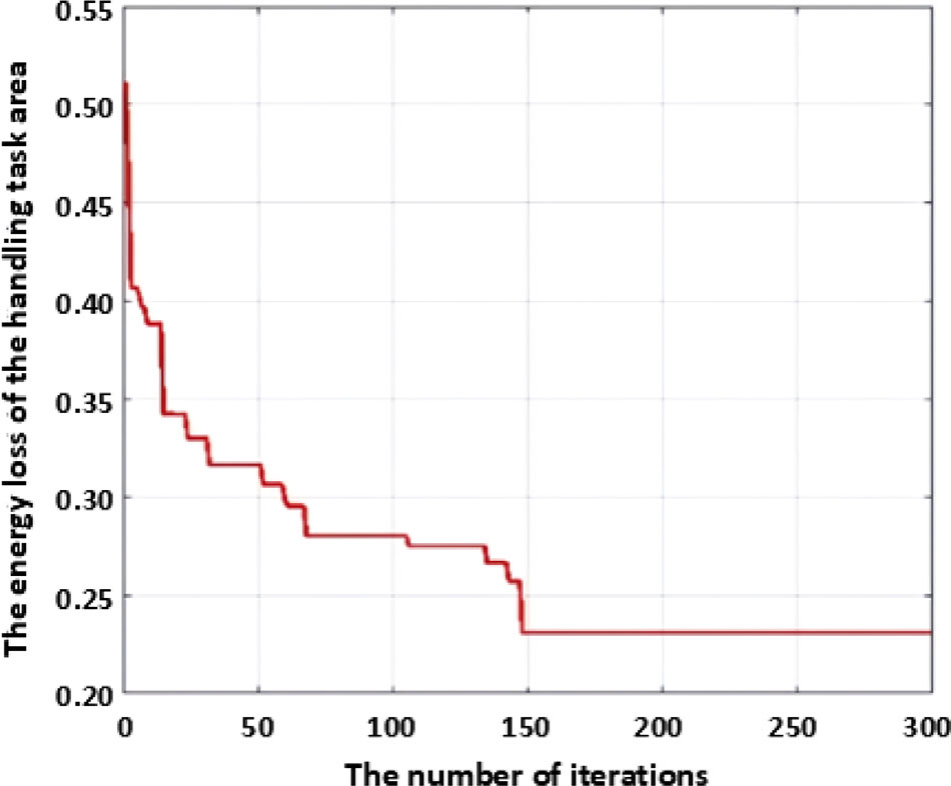
The fitness curve of the energy loss of the handling task area using the improved whale algorithm.

**FIGURE 9:**
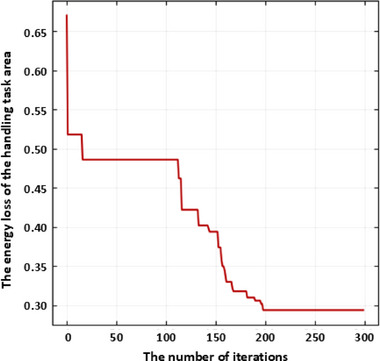
The fitness curve of the energy loss of the handling task area using the traditional whale algorithm.

The kinematic parameters of the six‐axis industrial robot (IRB140; ABB) are shown in Table 2. Take *n_w_* = 2000, and then solve the layout design based on the improved whale algorithm. Set the population size of whales to 30 and the maximum number of iterations to 300. The fitness change curve obtained is shown in Figure 8. It can be seen that when the number of iterations is 152, the fitness converges. And the optimal layout position is at the point (157.27, −313.59, 266.13). The overall average energy loss of the optimized handling task area is 0.2309. To verify the superiority of this method, a comparative experiment is conducted using the traditional whale algorithm, which does not introduce nonlinear convergence factors, to solve for the optimal layout scheme. The iterative result is shown in Figure 9. From Figure 9, it can be seen that as the number of iterations approaches 200, the curve converges. At this point, the energy loss of the six‐axis industrial robot reaches 0.2917. The optimized energy loss decreased by 20.84% compared to before optimization, successfully demonstrating the effectiveness and superiority of the method proposed in this paper.

**TABLE 2:** Kinematic parameters of the six‐axis industrial robot (IRB140; ABB).

Joint no.	1	2	3
Maximum angular velocity 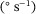	200	200	260
Maximum angular acceleration 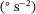	150	120	180
Maximum moment (Nm)	180	200	280

**FIGURE 10:**
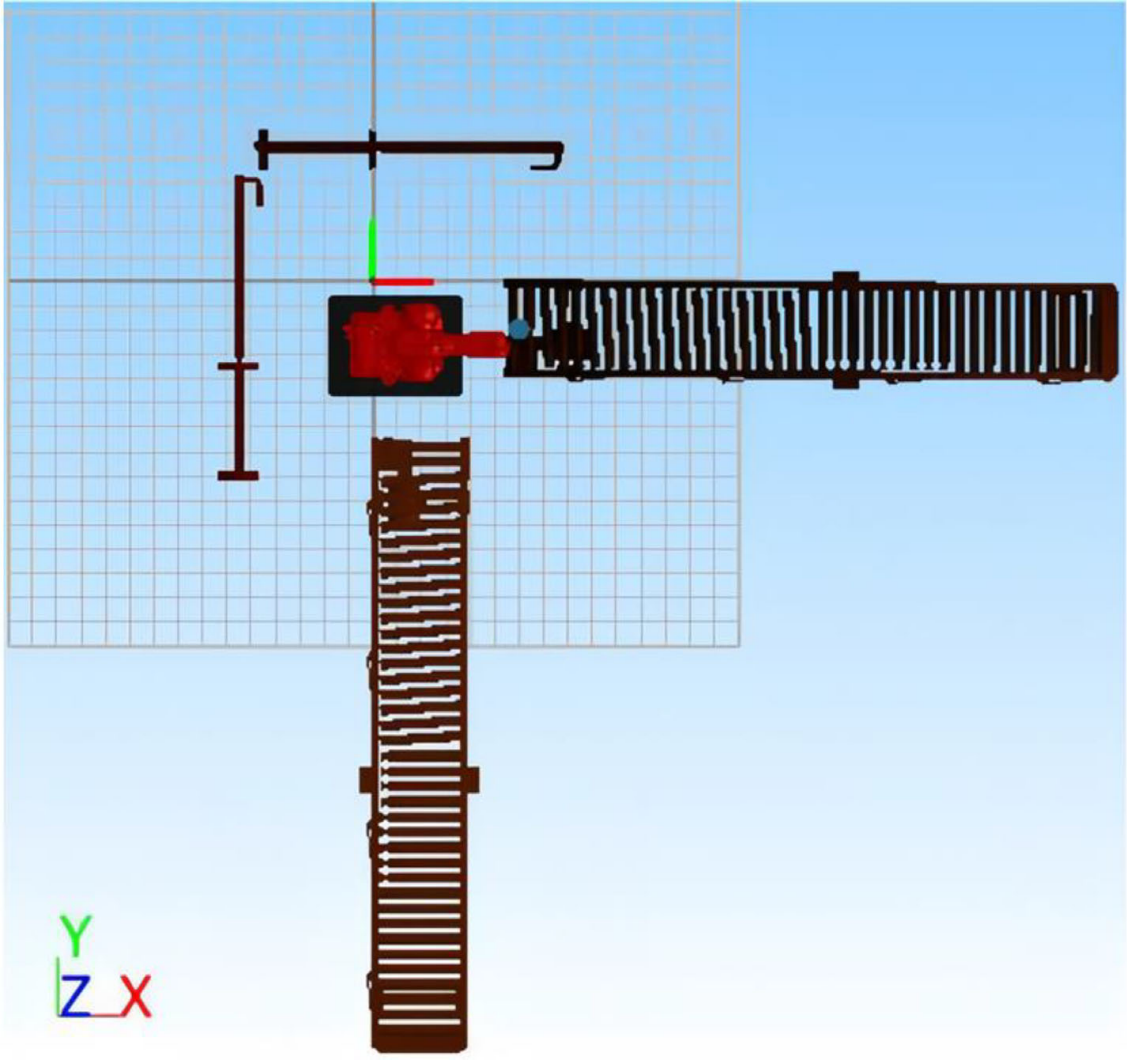
The optimized layout of the six‐axis industrial robot (IRB140; ABB).

**FIGURE 11:**
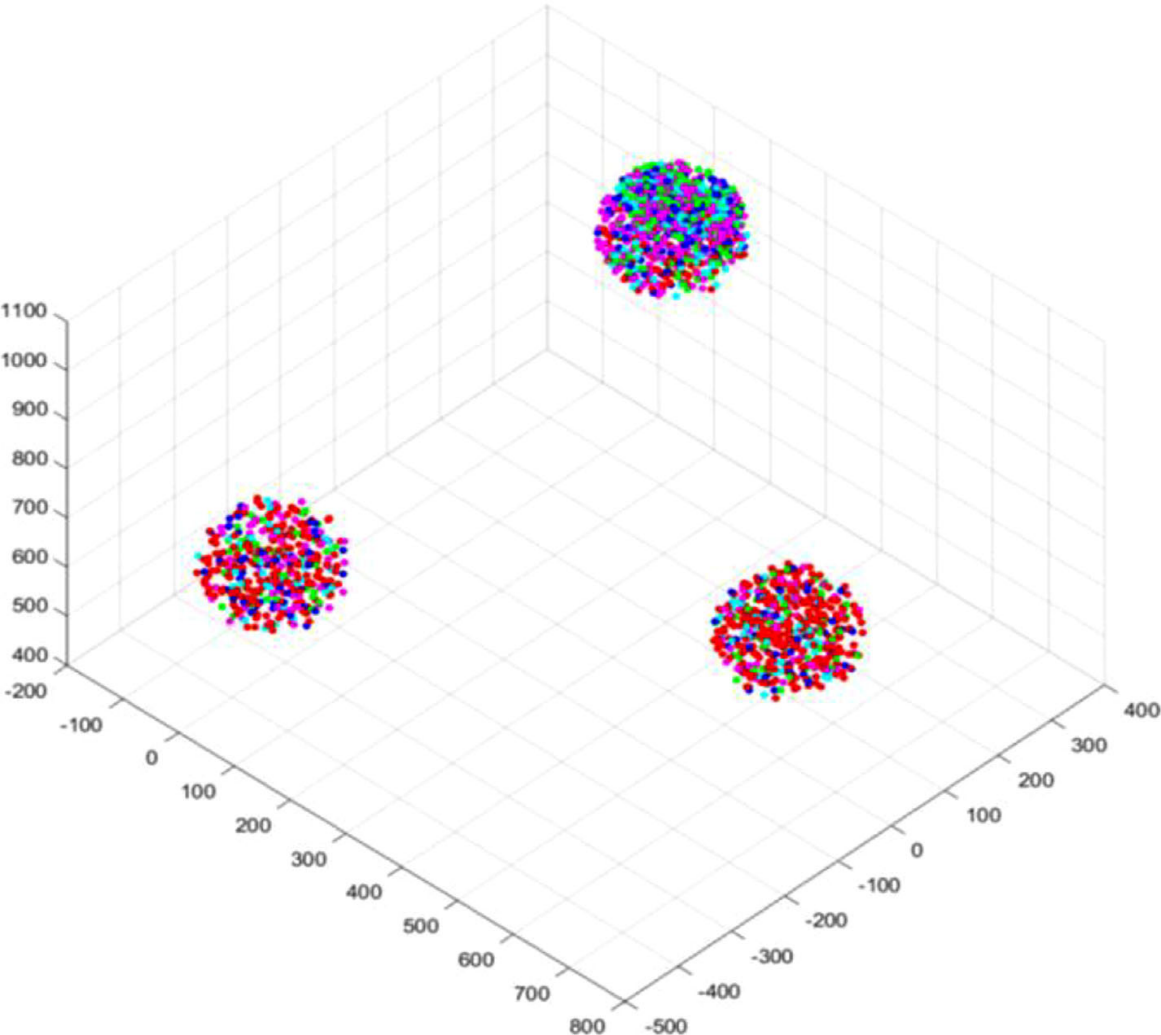
The energy loss of the sphere regions under the optimized layout.

Considering that the positions of industrial robots in actual work are all integers as far as possible, especially in the vertical direction, therefore, take one point in the workspace near the optimal layout position for the energy loss. The point (160, −315, 265) is used as the approximate optimal layout point. The regional energy loss at this point is 0.2573, which is fully enough to complement the handling task. The joint configuration of each point obtained from the optimal layout position is substituted into Equations (12) and (13) for verification, and it is found that it meets the requirements of the time constraint. The optimized layout position is shown in Figure 10. The energy loss of the sphere regions at the corresponding Cartesian workspace interpolation points is shown in Figure 11.

## 6 Conclusion

The application of industrial robots in manufacturing has contributed to the better management of cost, performance, and sustainability issues. Using industrial robots in manufacturing is beneficial to achieve sustainability and adaptability for Industry 5.0. However, unreasonable and complex spatial layout will reduce the flexibility of industrial robots and increase the energy consumption. Based on this, this paper creatively proposes a method to obtain the optimal layout scheme with the goal of minimizing the energy loss. First, the reachable workspace of the six‐axis industrial robot is solved, and the feasible workspace under interference constraints and time constraints is analyzed. Then, the operability and the minimum singular value are utilized to calculate the energy loss of the reachable workspace, and the reachable workspace is divided based on the energy loss. Next, the whale algorithm is improved to obtain the optimized layout of the industrial robot, which has the lowest regional energy loss. Finally, the case of the recliner's production line is provided and the proposed method successfully obtains the optimal layout. After optimization, the energy loss has been reduced from 0.2917 to 0.2309, a decrease of 20.84%. Proof by facts, the proposed method is economic and environmentally friendly which plays an important role in the layout optimization and could acquire a lot of attention in practical applications.

However, there are still some limitations in our research and we will work out them in future research: the energy consumption caused by the trajectory needs to be considered, and a novel multi‐objective optimization method with high efficiency and accuracy needs to be developed to fit the actual scene more closely.

## Author Contributions

**Kaiyue Cui**: conceptualization, methodology, software, validation, formal analysis, writing – original draft, visualization. **Yixiong Feng**: conceptualization, methodology, resources, supervision, project administration, funding acquisition. **Zhaoxi Hong**: conceptualization, methodology, writing – original draft. **Zhiwu Li**: conceptualization, methodology, writing – original draft. **Fathollahi‐Fard Amirmohammad**: methodology, revision and polishing of manuscript. **Zengwei Ji**: software, validation, visualization. **Jianrong Tan**: resources, supervision, project administration, funding acquisition. All authors read and approved the final manuscript.

## Conflicts of Interest

The authors declare no conflicts of interest.

## Data Availability

No data was used for the research described in the article.
